# Comparative study of virtual and face-to-face training methods on the quality of healthcare services provided by Kermanshah pre-hospital emergency staff (EMS): randomized educational Intervention trial

**DOI:** 10.1186/s12909-022-03277-y

**Published:** 2022-03-25

**Authors:** Reza Farahmand Rad, Akram Zolfaghari Sadrabad, Reza Nouraei, Alireza Khatony, Homayoon Bashiri, Arezoo Bozorgomid, Shahab Rezaeian

**Affiliations:** 1 Clinical Research Development Center, Imam Reza Hospital, Kermanshah University of Medical Sciences, Kermanshah, Iran; 2 Department of Emergency Medicine, Kermanshah University of Medical Sciences, Kermanshah, Iran; 3 Shohada Hospital, Kermanshah University of Medical Sciences, Kermanshah, Iran; 4 Infectious Diseases Research Center, Health Institute, Kermanshah University of Medical Sciences, Kermanshah, Iran

**Keywords:** Virtual training, Face-to-face training, Emergency medicine, Clinical skills

## Abstract

**Background:**

Emergency medical centers are globally one of the most important pillars of pre-hospital care. The most important purpose of this system is to provide satisfactory services in the shortest possible time and in accordance with the modern scientific standards of the world. The present study aimed to compare the effect of virtual and face-to-face training methods on the quality of service provided by Kermanshah pre-hospital emergency personnel, Iran**.**

**Methods:**

This was a randomized educational intervention trial performed among the staff of Kermanshah Emergency Medical Center. Individuals were randomly divided into two training groups of virtual and face-to-face. Participants in the face-to-face group received slides, lectures, and practical work with moulage for 6 h a day. Subjects were taught the four skills of intubation, laryngeal mask airway (LMA), cardiopulmonary resuscitation (CPR) and attenuated electrical device (AED). Participants in the virtual group received the same content in the form of a training video on CD with a full explanation of the project's objectives. Pre- and post-test scores of participants were compared within and between the groups by Stata 14.0 software.

**Results:**

Eighty-seven individuals were participated in the study, 43 of whom were assigned to the face-to-face training group and 44 to the virtual training group. There was no significant difference between the two groups in terms of work experience and educational level (*P* > 0.05). Post-training scores in both groups were significantly higher than pre-training in the four skills (*P* ≤ 0.005). After adjusting for educational level and work experience, however, the quality of CPR, intubation, and AED was higher in the face-to-face training group than in the virtual group. However, the increase in the mean score of LMA in the virtual training was not significantly different than that of the face-to-face training group.

**Conclusion:**

The results of our study showed the same efficacy of both face-to-face and virtual methods in improving the performance of personnel in tracheal intubation, LMA, CPR and AED shock skills. E-learning methods can be used as a complement to face-to-face methods in education.

## Background

Iran ranks fourth among the countries at risk for natural disasters. Of the 40 types of natural disasters observed in the world, 31 occur in Iran with an emphasis on earthquake in comparison to other hazards [1]. Also, with the increase in the number of motor vehicles, the number of fatalities due to accidents has increased and has become a threat to health systems in developing and developed countries [2]. Pre-hospital emergency system plays a major role in providing healthcare services and is at the forefront of dealing with patients and casualties caused by these accidents [3]. Therefore, its employees must have precise information and updated training. Because when they faced with life-threatening conditions outside the hospital, should be able to limit mortality by timely diagnosis and treatment.

According to research, sustained educational programs play an essential role in enhancing the knowledge and ability of medical staff [4, 5]. These programs are divided into two categories: face to face and virtual, each having its own advantages and disadvantages. The benefits of the face-to-face approach include the presence of the learner in the actual situation of instruction, the opportunity to see live teacher activities and instruction, and the opportunity for question and answer between learner and teacher. Disadvantages of the face to face method include the cost of organizing programs, the difficulty of providing the conditions, the inability of some learners to attend due to occupational or distance restrictions, the inability of all learners to participate actively, and the inability to provide this training to larger groups. However, virtual education is cheap and easy to reproduce, has no spatial limitations, is more viable in times of scarcity of human resources (lecturers or learners), can accommodate larger demographic groups, and is easier to be held compare to the face to face method. Alongside these benefits are factors that may reduce the effectiveness of this method. These include lack of virtual education equipment, lack of skill in using these equipment, confounding factors in the delivery of training (such as noise pollution on site and disruption of learner focus), and lack of live learning environment for learners [6, 7].

Virtual training is increasingly used in medical education such as nursing students’ practical skills, the teaching of oral pathology and anatomy, radiology, parasitology, etc. [8–12]. Many authors have expressed positive role of this approach in education such as reducing training costs, unlimitedly repetition of the same training, time flexibility, comfort and lack of transportation worries [13]. Examples exist within the literature which highlighted the effectiveness and values of both methods in teaching system [14, 15]. Nevertheless, a number of studies have reported that one of the virtual or traditional methods has been more effective than the other [16–18]. Given the inconsistency in the research findings and the sparsity of pre-hospital staff workplaces in Kermanshah Province and the need for regular sustained educational programs for them, the present study aimed to compare the effect of virtual and face to face training methods on the quality of service provided by Kermanshah pre-hospital emergency personnel.

## Methods

### Study design

This randomized educational intervention trial study was conducted from 22 December to March 2017 in Kermanshah University of Medical Sciences, Iran.

### Study hypothesis

The face to face teaching method has a greater impact on the quality of pre-hospital services than the virtual method.

### Sample and sampling method

The study population included all the pre-hospital personnel in Kermanshah Province. Sample size was calculated at 88 individuals based on Shaw’s study [19] with a first-order error of 0.05 and 80% power. The final sample size was estimated to be 98 considering 10% attrition. In this study, all pre-hospital staff were surveyed, some of whom were excluded from the initial study due to not meeting the inclusion criteria. The inclusion criteria included consent to participate in the study, computer literacy (based on individual statements), access to a computer, and employment in the Emergency Department of Kermanshah University of Medical Sciences.

**Fig. 1:**
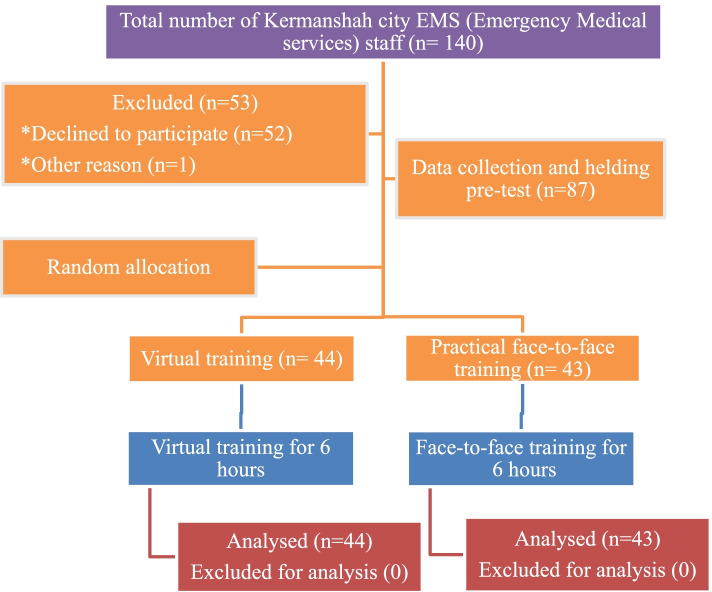
Flow chart of the study

 Finally, 87 eligible individuals were randomly divided into two groups (1:1 ratio) via sealed opaque envelopes with labeled 1 to 87. The individual’s training group was allocated when the envelope was opened (Fig. 1). No exclusion criteria were considered for this study.

In Iran, there is a lack of a protocol based on scientific evidence for evaluating the adequacy of therapeutic interventions. To this end, information related to American Heart Association's standard protocols were drawn and then evaluated in a meeting with the respective experts. Afterwards, standard questionnaire were designed on that basis. This questionnaire tapped into the four areas of tracheal intubation (4 questions), laryngeal mask airway (LMA) (9 questions), cardiopulmonary resuscitation (CPR) (12 questions) and attenuated electrical device (AED) (6 questions). The Cronbach's alpha coefficient was used to check the reliability of questionnaire by those four areas (The Cronbach's alpha was for LMA=0.76, CPR=0.82, AED=0.89, intubation=0.70). Initially, participants' skill level in providing pre-hospital services was assessed in person using an American Heart Association checklist by an emergency medicine specialist (third author) for three days at the emergency medical center. The participants' pre-test scores were recorded in the study.

### Intervention

Intervention in the face-to-face group in the four areas of tracheal intubation, LMA, CPR, and AED was performed in the form of lecture, slide show, and film and practical work for 6 h a day. The intervention was repeated the next day to allow for the enrollment of the participants who were unable to participate in the training on the first day due to occupational restrictions. The training was held in the Clinical Skills Hall of the Imam Reza Hospital, Kermanshah University of Medical Sciences, Kermanshah, Iran.

In the virtual training group, all of the above contents were recorded as a lecture, slide show, and film in collaboration with the Education Development Center of University of Medical Sciences (EDC), and then implemented on a CD. The CDs were given to the virtual training group by the emergency medicine center. The two groups' training programs were provided by an emergency medicine specialist (first author) with the same content. Based on the previous valuable literature [20], one month after the completion of the training, the participants' proficiency was re-assessed as a face-to-face test, such as a pre-test. The test was performed in both groups on a moulage for three days at the emergency medicine center by the person who performed the pre-test using a checklist.

Due to the nature of the study, no blinding was performed. To encourage these individuals to continue working until the end of the project, measures such as assigning re-training points to both virtual and face-to-face training groups were undertaken.

### Statistical analysis

Descriptive indices including frequency and percentage (for qualitative variables) as well as mean and standard deviation (for quantitative variables) were used to describe the data. To analyze the data, the Chi-square test was used to compare the qualitative variables between the two groups. In order to compare the quantitative variables between the two study groups, independent t-test (in case of normal distribution of the data) was used. In order to compare pre-test and post-test scores in each group, paired t test was applied. Analyzes were performed using Stata 14.0 software. The significance level was defined as 0.05.

## Results

The flowchart of our study population is shown in Fig. 1. As shown, 53 of 140 participants were excluded from the study for personal reasons. A total number of 87 EMS personnel participated, 43 (49.43%) in the face-to-face group and 44(50.57%) in the virtual group. All the participants in this study were male, given that pre-hospital emergency technicians are largely male in Iran. There was no significant difference in the work experience, age and educational level between the two groups (Table 1). Table 2 shows mean pre-test scores of the participants of two groups in the four studies skills (tracheal intubation, LMA insertion, CPR, and AED use). As seen, no significant difference was observed using independent t-test between the two groups regarding pre-test scores (29.0 ± 6.1 for the face-to-face group vs 28.4 ± 5.1for the virtual group; *P* = 0.613).

**Table 1:** Baseline demographic characteristics of the study participants

Variables	Face-to-face group	Virtual group	*P*-value
Age^a^	34 ± 7	34 ± 6	0.85
Job Experience (month)^a^	146.0 ± 75.0	134.3 ± 67.5	0.446
Education (%)			
Diploma and BA	14 (32.6)	20 (45.5)	0.218
BS and MSc	29 (67.4)	24 (54.5)	

^a^Values are expressed as Mean ± SD

**Table 2:** Comparison of the mean pre-test scores of emergency medical service (EMS) staff in the two study group

Variable	Face-to-face group	Virtual group	*P*- value
CPR	10.3 ± 2.1	10.5 ± 1.5	0.571
Tracheal intubation	7.4 ± 1.8	7.5 ± 1.6	0.874
LMA	6 ± 2.1	5.4 ± 2.1	0.213
AED	5.3 ± 1.3	5.0 ± 1.7	0.325
Total	29.0 ± 6.1	28.4 ± 5.1	0.613

Values are expressed as Mean ± SD

As it is shown in Table 3, in comparison of mean score differences in each skill, an increase in the mean score in the tracheal intubation, CRP, and AED was seen in face-to-face group in comparison to virtual group. Nevertheless, increase in score of tracheal intubation was seen in the virtual group in comparison to face-to-face group, although it was not statistically significant (*P* = 0.538). Also, the findings of the research showed that both groups had significantly higher total score and scores of each skill in the post-test (*P* ≤ 0.005) (Table 3).

**Table 3:** Comparison of the mean of emergency care methods before and after the intervention by the study groups

Variable		Face-to-face group	Virtual group	Mean difference^a^	*P*- value
CPR	Before	10.3 ± 2.1	10.5 ± 1.1	+ 0.08 ± 0.3	0.813
	After	11.6 ± 1.2	11.8 ± 0.8		
	*P*-value	< 0.001	< 0.001		
Tracheal intubation	Before	7.4 ± 1.8	7.4 ± 1.6	+ 0.11 ± 0.09	0.266
	After	8.8 ± 0.8	8.8 ± 0.6		
	*P*-value	< 0.001	< 0.001		
LMA	Before	6.0 ± 2.1	5.4 ± 2.1	-0.21 ± 0.3	0.538
	After	7.9 ± 1.7	7.8 ± 1.6		
	*P*-value	< 0.001	< 0.001		
AED	Before	5.3 ± 1.4	5.0 ± 1.7	+ 0.06 ± 0.1	0.559
	After	5.8 ± 0.7	5.7 ± 1.1		
	*P*-value	0.005	< 0.001		
Total	Before	29.0 ± 6.1	28.4 ± 5.1	+ 0.13 ± 0.4	0.774
	After	34.1 ± 3.7	34.2 ± 3.4		
	*P*-value	< 0.001	< 0.001		

^a^Mean difference adjusted for education, job experience and score for each item of emergency care methods before the intervention

Values are expressed as Mean ± SD

## Discussion

A worldwide shortage of health professionals in the health care system is an age-old problem and has negative effects on patient outcomes and quality health care. The development of virtual medical education courses can provide a lot of educational opportunities for students in low- and middle-income countries and reduce this shortage [21].

In this present study, we found no significant difference between virtual training group and face-to-face training group in all skills (CPR, tracheal intubation, LMA, and AED) and both methods appear to be effective. Furthermore, all participants in face-to-face and virtual learning methods completed this study that can reflect their willingness to use these two methods. Despite different study populations, our findings are in line with the study of Khatony et al. (2009) who did not find significant difference between the electronic and traditional educational methods in learning the principles of caring HIV positive patients [22]. According to the findings of Mahadevan’s research (2018), there was no significant difference between the online and traditional classroom course in knowledge gain among Ugandan medical students participating on emergency medicine [23]. Aminizadeh et al. (2019) noted that E-learning systems can be useful in subjects that theoretical teachings are more prominent in emergency intermediate technician. The results of their study showed that here were significant differences between the practical test scores (CPR, familiarity with the equipment, and proper patient transportation methods) in both the electronic and traditional methods, in all the skills except triage [17].

Virtual training is a new environment for learning that is able to overcome traditional education limitations. This method has no time or space limitations and is accessible to anyone who has a computer, tablet or Smartphone [24]. Of note, in our study, of 140 eligible EMS staff, only 87 subjects participated in workshop. Individuals stated that shift working and busy schedule were the causes of their non-participation. Accordingly, we propose that instructors use the virtual education method as an alternative source for training especially for people who can't afford the time to take real courses.

Although, there was no significant difference between the practical tests scores in both the virtual and face-to-face training methods, practical group scored better than the virtual training group in all skills expect LMA when the post-test scores were compared. However, participating in workshop not only allows learner to have a one-on-one experience with the instructor and fail in a safe situation but also exposes them to practical skills that can be beneficial in which they will be working.

In the current research, it was shown that both groups had significantly higher results in the post-test, which reflects improving participants’ knowledge and skills performance through participating in workshop. Ghiyasvandian et al. in their study, similar to our results, showed that the experience of performing tracheal intubation correlated with successful tracheal intubation [25]. Therefore, it can be said that the training and practice by the EMS staff is effective in improving their skills in performing tracheal intubation. Similar to the present study, Swanson et al. conducted a study to investigate the effect of training on ability of the participants in performing tracheal intubation at pre-hospital setting. They reported that education with practical training reduces failed tracheal intubation rate and resulted in successful tracheal intubation at the first attempt [26]. Weisfeldt et al. stated that the correct and timely use of AED in patients before accessing hospital care saves lives of the patients and reduces the complications caused by cardiac arrest. They added that AED use training courses should be held continuously for pre-hospital EMS staff [27]. Behmadi et al. also conducted a study to investigate the effect of the two methods of teaching lecture and virtual in teaching start triage lessons in emergency medical students. They concluded that that virtual can improve knowledge in undergraduate emergency student's education, but it is not superior to than traditional educational methods [28].

It is worthy to note that in Iran, the EMS personnel have different educational background such as nursing, operating room, anesthesia, and medical emergency technicians; therefore, they don't have the same level of knowledge and experience for pre-hospital care. So, holding training courses could provide the opportunity to practice and repeat and improving the skills and knowledge of the emergency staff.

### Limitations

Our study had some limitations such as sensitization of participants in both groups from intervention to post-test, use of related resources such as books and the Internet, communication of participants in work shifts and sharing opportunities, which could not be controlled by the researchers. In the virtual group, the participants received training as a CD containing the curriculum, and we had to trust them to track their use of the educational content; web-based virtual training programs could be used to solve this problem to control the presence of people in the training session.

## Conclusion

Our results reflected the same effectiveness of both face-to-face and virtual methods in improving personnel performance in AED, tracheal intubation, LMA and CPR skills, so in cases where providing face-to-face training conditions is difficult, especially when the training is more theoretical, it is suggested to use virtual training methods as a complement to in-person training. Future studies are recommended to be performed in other healthcare groups such as nurses and operating room technicians. It is also suggested to compare the impact of CD-based methods with web-based methods.

## Data Availability

The datasets used and/or analyzed during the current study are available from the corresponding author on reasonable request, subject to ethical approval.

## Abbreviations

EMS: Emergency medical services
LMA: Laryngeal mask airway
CPR: Cardiopulmonary resuscitation
AED: Automatic external defibrillation
CME: Continued medical education
